# Exploring Motives Behind Ideal Melanoma Survivorship Care Plans With Multiple Stakeholders: A Cocreation Study

**DOI:** 10.2196/55746

**Published:** 2025-01-02

**Authors:** Nadia Christina Willemina Kamminga, Marjolein Lugtenberg, Julia Annabel Van den Broek, Tamar Nijsten, Marlies Wakkee, Kasia Tabeau

**Affiliations:** 1 Department of Dermatology Erasmus MC Cancer institute University Medical Center Rotterdam Rotterdam Netherlands; 2 Department Tranzo Tilburg School of Social and Behavioral Sciences Tilburg University Tilburg Netherlands; 3 Erasmus School of Health Policy & Management Erasmus University Rotterdam Rotterdam Netherlands

**Keywords:** cocreation, survivorship care, psycho-oncology, supportive care, motives, melanoma, cancer survivor, melanoma care

## Abstract

**Background:**

Survivorship care plans (SCPs), ie, personalized health care plans for cancer survivors, can be used to support the growing group of melanoma survivors throughout their disease trajectory. However, implementation and effectiveness of SCPs are suboptimal and could benefit from the involvement of stakeholders in developing a user-centered design.

**Objective:**

The aim of this study was to identify the ideal SCP for patients with melanoma in terms of functions and features to be included according to different stakeholders and to explore their underlying motives.

**Methods:**

In total, 3 cocreation sessions were organized with mixed samples of stakeholders, ie, patients with (a history of) melanoma (n=4), health care providers (HCPs) active in melanoma care (n=3), and IT specialists active in hospital IT departments (n=6). They were invited to compose their ideal melanoma SCP based on potential functions and features identified from prior qualitative research. These functions and features belonged to one of the four main categories of survivorship care (SSC): (1) information and education, (2) identification and treatment, (3) oncological follow-up, and (4) coordination. Participants were invited to explain their motives for including functions and features. Ideas were shared between stakeholders, and interaction was promoted. Descriptive statistics were used to determine the ideal SCP per stakeholder group. To analyze underlying motives, all cocreation sessions were audio-taped, transcribed verbatim, and analyzed in a thematic content analysis.

**Results:**

With regard to their ideal SCPs, all stakeholders added functions from all 4 SSC categories. Patients assembled a rather compact SCP with category 2 on *identification and treatment* being most important. Both HCPs and IT professionals constructed a somewhat larger SCP, with category 3 on *oncological follow-up* being the most important aspect and HCPs also focusing on category 4 on *coordination*. As for the motives behind their ideal SCP compositions, patients predominantly added functions based on their personal experiences or experiences from fellow patients, whereas both HCPS and IT professionals based their compositions primarily on their respective areas of expertise: HCPs related their additions to their roles as medical practitioners; for example, in providing a complete treatment plan and obtaining informed consent, while IT professionals’ contributions were mainly influenced by feasibility and privacy concerns.

**Conclusions:**

This cocreation study provides insights into stakeholders’ ideal melanoma SCP and the motivations behind them. Considering the diversity in both the preferences and underlying motives regarding SCP composition between patients, HCPs, and IT specialists, it is crucial to develop a broad SCP that extends beyond traditional SCP content, emphasizing personalization. In addition to continued stakeholder involvement, efforts should be focused on addressing potential feasibility and privacy issues to ensure the SCP meets both patients’ and HCPs’ needs.

## Introduction

In recent years, the prognosis of melanoma, one of the most aggressive forms of skin cancer, has significantly improved due to advancements in innovative treatments such as immunotherapy and targeted therapy [1]. With an estimated worldwide total of 325,000 new cases in 2022, increasing to an expected total of 510,000 new cases in 2040 [2], this results in an expanding cohort of melanoma survivors, ie, individuals living with or beyond melanoma [3]. To ensure that patients get the necessary support throughout their treatment trajectory and assist them in resuming life thereafter, it is important to provide them with effective survivorship care (SSC) [4].

SSC can be divided into four main categories [4,5], namely (1) information and education, (2) identification and treatment, (3) oncological follow-up, and (4) coordination (Textbox 1 [4,5]). Survivorship care plans (SCPs), personalized health care plans for cancer survivors, have an important role in the delivery of SSC, traditionally mainly regarding categories 1 and 3 of SSC (Textbox 1) [4]. However, notwithstanding their potential benefit for both patients and health care providers (HCPs) and the recommendation of their use in clinical guidelines, the present implementation and effectiveness of SCPs seem to be suboptimal [6-8]. Until now, most SCPs have been static, paper-based documents, while patients have shown a preference for dynamic, electronically accessible formats that permit alterations and accessibility for all stakeholders [8]. Personalization, an essential element of SCPs, has often been overlooked, despite evidence emphasizing the importance of tailoring SCPs to accommodate the diverse information needs among patients [9]. However, these findings are mostly based on evaluations of the SCP subsequent to its implementation and based on the feedback of 1 single type of stakeholder. Indeed, while stakeholder engagement seems critical for effective implementation [10], involvement of key stakeholders like patients and HCPs during SCP development has been limited until present [8,11]. The specific needs of patients with melanoma and their HCPs regarding the content of melanoma SCPs have been explored previously [12], which showed that while both stressed the importance of adequate information throughout the disease trajectory and personal oncological follow-up, different opinions existed regarding psychosocial support and coordination of care. However, the reasons why they consider these elements important and why their opinions differ remain unknown. Thus far, only the needs of users have been investigated, with developers’ perspectives yet to be examined, even though this could provide valuable insights into the feasibility of desired content.

An approach to integrating the diverse perspectives of all stakeholder groups, ie, patients, HCPs, *and* IT professionals (future developers), is to engage them in a cocreation process [13,14] that encourages their direct involvement in SCP development. Cocreation allows stakeholders to be active partners in the development of innovations, as opposed to objects of study, resulting in products and services that people want and need [15,16]. Therefore, the objective of this study is to investigate the ideal SCP in terms of functions and features to be included per stakeholder group and to explore the motivations behind these preferences through a cocreation process. The findings of this study will serve as a basis to design a user-centered, practically feasible SCP that is tailored to the needs of stakeholders and thereby more easily integrated in clinical practice.

**The 4 main categories of survivorship care.**

- *Information and education* about the disease, its treatment, and the possible early and late effects.
- *Identification and treatment* of the disease and therapy effects on all possible domains (ie, physical and psychosocial, including work- and insurance-related).
- *Oncologic follow-up* with surveillance for cancer progression, recurrences or second cancers.
- *Coordination* between all health care providers involved in the care process, to make sure the survivor’s health needs are met.

## Methods

### Setting

This study was part of a regional project in which a digital and personalized melanoma SCP will be developed that will be linked to the patients’ electronic health record and provided to patients from diagnosis onwards to help them deal with all disease and treatment-related impacts [17,18]. This project takes place in the region Rijnmond, the Netherlands, and forms a collaboration between 1 academic (Erasmus Medical Center) and 3 non-academic hospitals (Albert Schweitzer Hospital, Francicus Gasthuis & Vlietland, and Maasstad Hospital), in which, like internationally [7], SCPs are not yet routinely provided [6]. The project consists of multiple phases—from needs assessment to implementation—in which cocreation is used to develop an SCP that is adapted to all stakeholders’ needs [13].

### Study Design

In this study, qualitative research methods in terms of cocreation sessions were used to gain an in-depth understanding of the preferences of all stakeholders involved [19]. Conducting cocreation sessions allows both the end users, ie, patients and HCPs and developers, ie, IT specialists, to collaborate in the SCP design process to reduce the gap between research and implementation [13,14].

The COREQ (Consolidated Criteria for Reporting Qualitative Research) guidelines [20] were used in reporting this qualitative study.

### Participants and Recruitment

Eligible participants were patients with (a history of) cutaneous stage I-IV melanoma; HCPs involved in both primary, secondary, and tertiary melanoma care such as dermatologists, oncologists, surgeons, general practitioners, and nurse practitioners; and hospital-based IT professionals, for example, those working in organizational aspects of IT in health care and eHealth (future developers of the SCP). We aimed to recruit an equal number of patients, HCPs, and IT professionals to ensure a balanced cocreation session in terms of perspectives to be included and to explore the motives in these perspectives. All patients had to be treated in and therefore under follow-up in, and both HCPs and IT professionals had to be affiliated with one of the 4 participating hospitals. To select participants, first patients and HCPs that participated in prior qualitative research and/or had given consent to be contacted (again) for participation in a follow-up study were invited to participate. IT professionals as well as the remaining patients and HCPs were approached through the professional networks of the researchers. All potential participants received information about the study by email or by phone. Our aim was to recruit ±15 participants, in which we follow prior research (eg, Vandekerckhove et al [21]). In the end, 19 participants signed up, and based on their availability and eventual willingness to participate, a total of 3 mixed cocreation sessions with 4-5 participants were organized, with a total of 4 patients, 3 HCPs, and 6 IT specialists. No financial compensation was given for participation.

### Cocreation Sessions

Input for the cocreation sessions was based on prior in-depth qualitative research, in which SSC needs of a total of 50 patients with stage I-IV melanoma and 24 HCPs were explored [6,17,18]. In total, 23 interview- and 8 focus group transcripts were re-analyzed for the purpose of this study using Nvivo version 12/R1, to identify potential functions and features of a melanoma SCP. *Functions* represent the overarching areas in which support can be provided, while *features* are tools to deliver that support. First, all transcripts were coded to identify patients’ and HCPs’ needs regarding SSC (including SCPs) by 2 researchers (JB, a female medical master student and a female health care management master student), which was then checked and complemented by 2 other researchers (NK, a female medical doctor, and KT, a female academic researcher in health care innovation and cocreation). The resulting SSC needs were reformulated as 44 potential features of an SCP by the research team (first reformulation by JB and a female health management student, under supervision of NK and KT, which was then discussed in the multidisciplinary research team including NK, ML, JB, KT, and a medical student until consensus was reached). Subsequently, an exploratory literature review was conducted to assess completeness, which did not reveal any new features. Subsequently, the features were structured according to the 4 main categories of SSC [4,5], as presented in Textbox 1, and further divided into 14 potential functions. Using this classification, the 44 potential features of a melanoma SCP were presented to the participants of the cocreation sessions (see Multimedia Appendix 1).

As a result of the national restrictions placed in response to the COVID-19 crisis, the 3 cocreation sessions were held online via Zoom in May 2021. All sessions took approximately 90 minutes and were moderated by JB and NK (experienced in moderating group discussions) and a health care management student, who were all not directly involved in melanoma care.

During the cocreation sessions, a PowerPoint presentation was used to present potential functions and features, and participants were invited to individually create their “ideal SCP” by placing their preferred features in a box. Subsequently, participants had a plenary, semistructured discussion in which they were encouraged to share their ideas about the ideal SCPs by sharing their screens. They were invited to elaborate on their motives for (not) including certain functions and features, and discussions arose. At the end of the sessions, the moderators questioned the participants about under-discussed features. All cocreation sessions were audio-taped, and participants were invited to hand in their filled-in sheets.

### Data Analysis

**Figure 1:**
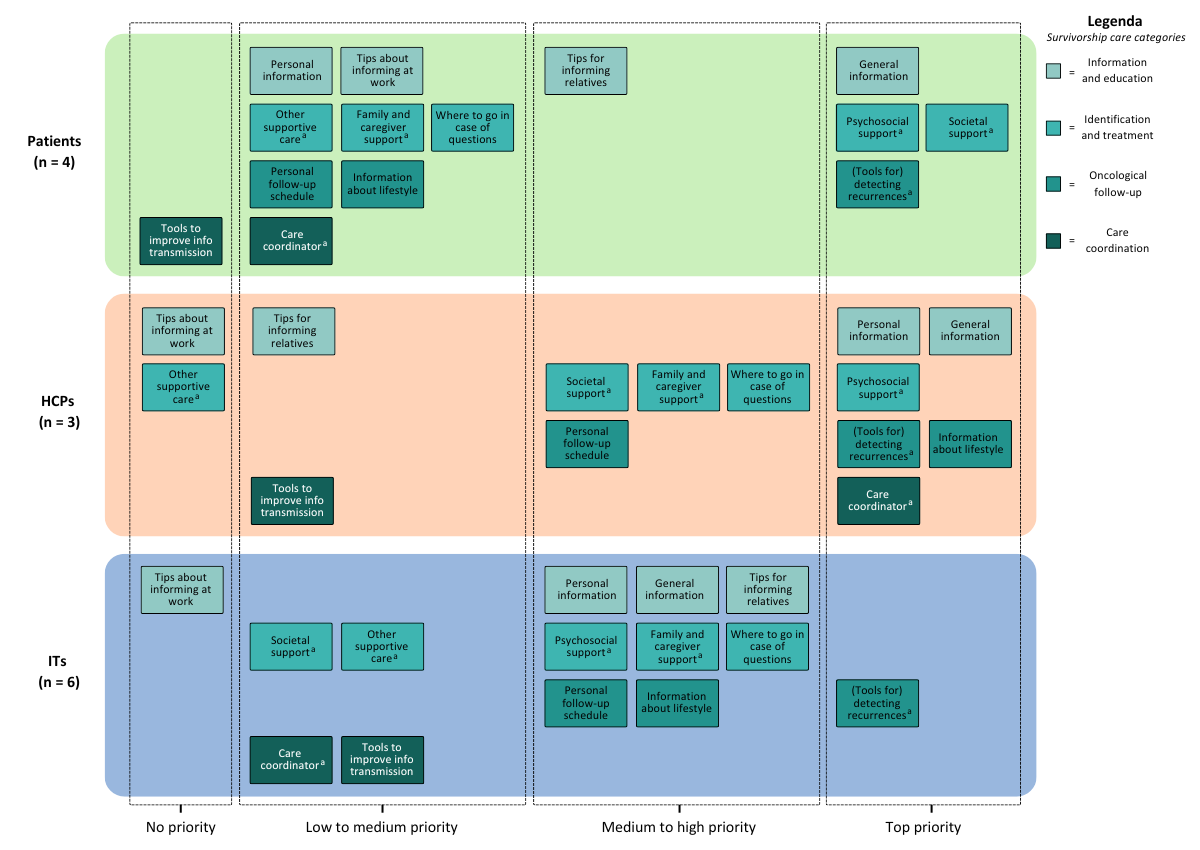
Overview of composed ideal SCP per stakeholder group in terms of functions added per main category of SSC. For an overview of all functions, including their corresponding features, see Multimedia Appendix 1. ^a^Information about and referral to reliable and up-to-date information and tools regarding this topic. HCP: health care provider; SCP: survivorship care plan; SSC: survivorship care.

All 3 audio-recordings were transcribed verbatim in anonymized form and were analyzed using Nvivo version 12/R1. Descriptive analyses were used, using Microsoft Excel, to determine the ideal SCPs of the different stakeholder groups based on the filled-in PowerPoint sheets and on comments they made during the sessions. For each function and feature, it was determined by how many percent of the participants they were included. Subsequently, all functions were categorized into “top,” “medium to high,” “low to medium,” or “no priority” based on the following criteria: 100% of respondents adding the function to their ideal SCP was defined as “top priority,” 50%-99% as “medium to high priority,” 1%-49% as “low to medium priority,” and 0% of respondents adding the function to their ideal SCP was defined as “no priority”; see also Figure 1. Based on this figure, we then described the ideal SCP par stakeholder group and the similarities and differences between them.

Second, underlying motivations of all participants for including functions and features in their ideal melanoma SCPs were analyzed in a thematic content analysis [22]. As a first step of the analysis, all transcripts were coded based on the functions and features within the 4 main categories of SSC, as previously mentioned (Textbox 1), by 2 researchers (JB and a health care management master student). This was checked and complemented by a third researcher (medical master student). Next, within these categories, underlying motives for composing the ideal SCPs were explored. Motives of each stakeholder group to include (or exclude) a specific function or feature in the SCP were openly coded by one researcher (JB), which was checked and complemented by a second researcher (NK). The next phase of analysis consisted of axial coding, in which the open codes were clustered in concept motives and links between motives and stakeholder groups were made in order to investigate the differences and resemblances between the ideal SCPs for patients, HCPs, and IT specialists. The resulting overview was discussed within the multidisciplinary research team until consensus was reached (JB, NK, ML, and KT).

### Ethical Considerations

This cocreation study was part of a larger project, of which the study protocol was submitted to, and approved by, the Medical Ethics Committee Erasmus MC. After reviewing the protocol, the committee concluded that the Medical Research Involving Human Subjects Act (Dutch abbreviation: WMO) did not apply to this study (MEC-2020-0197). Written informed consent was obtained from all participants involved in the cocreation sessions and they were informed that they could withdraw at any point during the study. Participants did not receive any compensation for participation in this study.

## Results

### Participant Characteristics

The characteristics of the 13 participants in the cocreation sessions as well as the compositions of all 3 sessions can be found in Table 1.

**Table 1:** Characteristics of participants per cocreation session.

Participant		Gender	Stakeholder	Experience or expertise	Care setting
**Session 1**					
	1	Female	Patient	Melanoma stage IV	Secondary or tertiary care
	2	Female	IT professional	Assistant professor in organizing aspects of IT in health care	Tertiary care
	3	Male	IT professional	Information management and IT advisor	Tertiary care
	4	Female	IT professional	Clinical informatician and data protection officer^a^	Secondary care
**Session 2**					
	5	Male	Health care professional	General practitioner	Primary care
	6	Female	IT professional	Information manager in research and innovation	Secondary care
	7	Female	IT professional	Clinical informatician: information advisor and architect, (application) implementation lead	Secondary care
	8	Male	Patient	Melanoma stage I/II	Secondary care
**Session 3**					
	9	Female	Health care professional	Oncological surgeon	Tertiary care
	10	Female	Health care professional	Oncological nurse practitioner	Secondary care
	11	Female	IT professional	Information manager	Tertiary care
	12	Male	Patient	Melanoma stage IV	Secondary or tertiary care
	13	Male	Patient	Melanoma stage IV	Secondary or tertiary care

^a^Having a partner with melanoma.

### Ideal SCPs and Underlying Motives

In Figure 1, an overview is provided of the composed ideal SCPs per stakeholder group, followed by an in-depth description of their underlying motives for (not) including certain functions and features. All results are discussed per category of SSC (Textbox 1), and all motives are provided from the participants’ perspectives.

#### Ideal SCPs

Overall, patients assembled the smallest SCP; for them, fewer functions were of medium to high, or top priority compared to the other 2 stakeholder groups. For patients, category 2 focusing on identification and treatment was most important. They primarily included general information and support for themselves, tips on how to inform their relatives, and information about (tools for) detecting recurrences. On the other hand, both HCPs and IT professionals constructed a somewhat larger SCP with more functions being of medium to high, or top priority, with category 3 focusing on oncological follow-up being the most important aspect. HCPs added more information about (personal) follow-up for the patient, including (tools for) detecting recurrences. Furthermore, they added both general and personal information, as well as support for both the patient and their family and caregivers. Additionally, they included information about a care coordinator. IT professionals, on the other hand, considered (tools for) detecting recurrences particularly important to add, and they included extensive information about the patient’s (personal) follow-up, both personal and general information, along with support for the patient and their family and caregivers.

#### Underlying Motives Per Stakeholder Group

For each stakeholder group, motives for (not) including certain functions and features are discussed below per main category of SSC and can be found in Table 2. For readability, results are provided on the function level, which are **bold**. Submotives are in *italics.*

**Table 2:** Overview of motives for (not) including functions and features from the 4 main categories of SSC in the ideal SCP, per stakeholder group.

Motives and submotives				PT^a^		HCP^a^		IT^a^
**Motives for including functions and features**								
	**Informing patients**							
		To meet patients’ information needs	1, 3		1, 2, 3		1, 2, 3	
		To provide understandable information	1		1		1	
		To provide reliable information	1		2		1	
		To help remember provided information	1		1			
		To obtain informed consent			1			
	**Helping patients deal with psychosocial issues**							
		To identify psychosocial issues			2			
		To alleviate patients’ concerns	3		3, 4		3	
		To provide support for or treat psychosocial issues	2		2		2	
		To help patients deal with lack of understanding of others	1				1^b^	
	**Improving patient empowerment**							
		To improve patients’ self-management skills	3		3		1, 2, 3	
		To support patients in decision-making			4			
	**Preparing and providing structure for patients**							
		To prepare for or raise awareness about what to expect	1		1			
		To prevent unnecessary consultations	3		2, 3		1	
		To provide structure for patients			3			
	**Empathizing with patients and their situation**							
		To empathize with (other) patients’ needs	2, 3		2		1, 2, 3	
		To take the patient’s context into account			1, 2, 4			
	**Meeting the relatives’ needs**							
		To meet relatives’ information needs			1, 2, 3		1	
		To meet relatives’ support needs					2	
	**Improving health and outcomes**							
		To improve melanoma outcomes			3		3	
		To improve health in general			3			
	**Providing integrated care**							
		To improve communication and collaboration between HCPs			4		4	
		To provide a complete treatment plan			2, 3		2	
		To provide an accessible contact point	2, 3, 4		2, 3, 4		2, 3, 4	
	**Relating to experiences and expertise**							
		Based on own experiences	1, 2, 3, 4				1, 2, 3, 4^b^	
		Based on experiences of others	2, 3				1, 2^b^	
		Based on expertise			1, 2, 3		1, 2, 3, 4	
	**Taking feasibility and privacy into account**							
		Function is feasible					1, 2, 3, 4	
		Function is important and this exceeds potential privacy issues					2, 3	
**Motives for not including functions and features**								
	**Preventing patient distress**							
		To prevent (irrelevant) information overload			1, 3		3	
		To prevent taking away hope	1				1^b^	
	**Preventing unnecessary development**							
		SCP is not the right medium			2		4	
		Similar tools already exist	3		3		3	
		Function is applicable in general, not for melanoma specifically					1, 3	
		Lack patient need for function	3					
	**Taking feasibility and privacy into account**							
		Function is unfeasible					2, 3, 4	
		Privacy issues are more severe than the importance of including the function and its relevance for patients					3	

^a^The numbers correspond with the main category of SSC to which the functions belonged and for which submotives were provided, namely (1) information and education, (2) identification and treatment, (3) oncological follow-up, and (4) coordination.

^b^Submotives for (not) including functions provided solely by an IT professional with a partner with melanoma.

#### Motives of Patients

The most important category for patients was category 2 (identification and treatment). Patients included **psychosocial support**, **societal support**, and **where to go in case of questions** because it would *help them* in *dealing with psychosocial issues*. Furthermore, they indicated that the SCP should contain information about and referral to (reliable and up-to-date information about) **psychosocial** and **societal support** because patients recognized the existence of such issues *based on their personal experiences* and because they *empathized with other patients’ (diverse) needs.*

Of course it’s all very individual.. One person might struggle with their mortgage, while another might not have financial issues due to their illness. […] And chatting with fellow patients for example, peer support, can be helpful for many people, although I personally don’t have the need for it.

Patient, male

Patients included **general information** and **tips for informing relatives** from the second most important category, namely category 1 (information and education), because they valued being *informed* and *prepared* and to help them *deal with their psychosocial issues*. They indicated that the ideal SCP should provide **general information** that is *reliable* and *understandable* since much of what is currently included in, for example, the patient portal is too medical and written in “doctor's language.” According to them, providing this information within the SCP could potentially prevent both patients and their relatives from searching for (incorrect) information on websites.

I did a lot of googling myself during the years I was under treatment, and at some point, you end up on really unpleasant websites where you're practically told that you'd be better off not living, and five years later, here I am. I found that very distressing, and it always made me very sad. So, I would like people to know how to find their way to the right information.

Patient, female

**Tips** or **information about informing relatives** could also *help them deal with psychosocial issues*, for example *with the (lack of) understanding of others.* A reason considered for not including functions from this category in their ideal SCP, such as specific parts of **general information**, was to *prevent getting distressed*; they emphasized not to add specific information, such as treatment effectiveness, because, *in their experience*, it *took away their hope.*

*Being informed, prepared, and empowered* were important motives to include **(tools for) detecting recurrences**, a **personal follow-up schedule**, and **information about a healthy lifestyle** from the third category (oncological follow-up), as was *dealing with their psychosocial issues*, which they did when *empathizing with other patients’ needs*. Furthermore, patients stressed that adding information regarding **(tools for) detecting recurrences** could *improve their self-management skills, alleviate their concerns*, and (thereby) *prevent unnecessary consultations*. Motives not to include functions from this category (eg, **information about a healthy lifestyle**) were that similar functions or tools already exist so that *unnecessary development could be prevented*.

Functions in the fourth and last category (coordination), like information regarding a **care coordinator**, although less important in their ideal SCP, were added *based on their own experiences* since they missed an *easily accessible contact point* during their disease trajectory*.*

#### Motives of Health Care Providers

HCPs added functions from category 3 (oncological follow-up) to *inform, prepare,* and *empower patients*. More specifically, they indicated that the ideal SCP should include information about/referral to **(tools for) detecting recurrences** to *meet patients’* as well as *relatives’ information needs*, *improve patients’ self-management*, and *alleviate their concerns.* Altogether, this could *prevent unnecessary consultations. Meeting patients’ information needs* and also *improving their melanoma outcomes and health in general* were reasons for adding **information about a healthy lifestyle**. Furthermore, HCPs indicated that offering this information is needed in order *to provide* patients with *a complete treatment plan*, which they did *based on their expertise*. However, others mentioned that *similar tools already exist* and therefore did not add this function to the SCP. A **personal follow-up schedule** including background information was added by HCPs to *meet patients’ information needs* and *to provide structure for them.* On the other hand, a reason for not including this background information was also mentioned, namely *to prevent (irrelevant) information overload.*

*Identifying and providing support for or treating psychosocial issues* were motives mentioned by HCPs for adding **psychosocial support** from category 2 (identification and treatment) to their ideal SCP. They did this *based on their expertise* and when *empathizing with patients’ needs*. Other reasons mentioned for adding this function were to *provide reliable information* (preferably by linking it to existing trustworthy resources) that is up-to-date and to *provide an accessible contact point* for patients. Furthermore, HCPs indicated that information regarding and/or referral to **societal support** should be added to meet patients’ *information needs* since questions regarding financial and work-related issues arise from diagnosis onwards. **Family and caregiver support** was added by the HCPs to *provide a complete treatment plan* for the patient and their loved ones and to *meet the relatives’ information needs*, the latter being important as heredity is a topic that elicits many questions. HCPs indicated that by better informing relatives, *unnecessary consultations* could be *prevented*. Lastly, **where to go in case of questions** was added by HCPs to *provide structure for patients* and to help them find the right person to turn to among the many HCPs involved in their illness and treatment journey.

That the patient actually has some idea of where to go for which question, so they don't get lost in the maze of various professionals.

Health care professional, male

Similarly, regarding category 4 (coordination), they added information regarding a **care coordinator** to *provide an accessible contact point* for patients and their relatives. They also did this *to alleviate patients’ concerns*, which is something that such a contact point could do*.* In addition, they considered this information important to ensure that the patient had a sense of having somewhere (or someone) to turn to for questions and uncertainties.

Functions from the first category (information and education) that were added to the ideal SCP of HCPs were both **personal** and **general information**, and they included them to *meet the patients’ information needs, to provide understandable information, to help patients remember provided information*, and *to support them in (treatment) decision-making.* They mentioned that patients often forget important **personal information** about their received diagnosis and treatment options, but also **general information regarding** what certain treatments (effects) entail, and they considered it important to assist them in retaining this information. In general, HCPs *based this on their expertise* and indicated they should provide this information in order *to obtain informed consent.*

You're supposed to tell them what you're going to do, what the side effects are, what the chances are that it will work; make it clear what the patient can choose. Only then can they give informed consent. So, whether you're going to perform surgery or provide immunotherapy, this is the information you must share.

Health care professional, female

#### Motives of IT Professionals

Category 3 (oncological follow-up) was the most important category in the ideal SCP of IT professionals. They added information regarding **(tools for) detecting recurrences** based *on their own experiences* and when *empathizing with patients’ needs.* Furthermore, *to improve patients’ self-management skills, to alleviate their concerns*, and *to provide an accessible contact point* were also mentioned as motives for including this function. IT professionals indicated that they thought patients could have worries and uncertainties around potential recurrences and that support regarding these worries, which is also *feasible*, should be provided to them. While *empathizing with patients’ needs,* they indicated that a **personal follow-up schedule** including background information should be added *to meet patients’ information needs*. However, *taking feasibility and privacy into account,* they mentioned that while they considered adding background information feasible, they foresaw several *privacy issues* for including a **personal follow-up schedule** that *were more severe than the importance of including and the relevance of* this information.

For privacy regulations, I deliberately left those things out because otherwise, it makes it quite challenging when it comes to an appointment calendar or something similar. If you manage it yourself, it's fine, but if it has to come through the hospital information system, then you have to integrate with that, and it becomes really tricky from an IT and privacy standpoint.

IT professional, female

They added **information about a healthy lifestyle** for patients with melanoma when *empathizing with* and *to meet their needs*, as well as *to improve melanoma outcomes*. Reasons for not including this information were that *having a healthy lifestyle is applicable in general, to prevent (irrelevant) information overload* and because *similar tools already exist*.

IT professionals added functions belonging to category 2 (identification and treatment) *based on their expertise* and, more specifically, because they considered them *feasible.* In addition, they added **psychosocial support** to *provide support for or treat psychosocial issues* and *to improve patients’ self-management*, which they *based on their own and others’ experiences.* They added **family and caregiver support** to *meet patients’ and relatives’ information and support needs* and *to provide a complete treatment plan*. Some included information about **where to go in case of questions** to provide clarity for patients; although some of them questioned its feasibility, they thought *its importance and relevance for patients outweighed the potential privacy issues.*

I think that if you can have patients fill in information like, 'I have this general practitioner, I go to that hospital with that specialist, and these are the other health care providers involved,' and alongside that, a general guide saying, 'For these kinds of questions, contact your GP,' I think that can provide more value. But when you want to retrieve that information automatically, it becomes challenging.

IT professional, female

For others, these *potential issues* made them consider this function *unfeasible*, and they did not add this to their ideal SCP.

*Informing*, *preparing*, and *empoweringpatients* were reasons mentioned by IT professionals to include functions like **personal** and **general information** from category 1 (information and education). They added them *to meet* both *patients’ and relatives’ information needs, to provide understandable* as well as *reliable information*, and to, at the same time, *help patients deal with the lack of understanding of others*. **Tips to inform relatives** were added by the IT professionals to *meet relatives’ information needs* and to *help patients deal with a lack of understanding of others*, which they did *based on the experiences* they gained *from others*. They considered adding these functions (**personal** and **general information** and **tips to inform relatives**) *feasible* as *based on their own expertise* and when *empathizing with patients’ needs*. Reasons for not adding functions like **general information** included considering them *not applicable to melanoma specifically* and to prevent this general information from *taking away hope*.

IT professionals considered category 4 (coordination) least important. Nevertheless, they added functions (**care coordinator** and **tools to improve information transmission**) from this category *based on their own and others’ experiences* and based on *their expertise.* They also provided motives for not including functions from this category; they indicated that according to them, the *SCP is not the right medium* to include **tools to improve the information transmission between HCPs**, a function that they also considered *unfeasible* to realize*.*

## Discussion

### Principal Findings and Comparison With Prior Work

The aim of this study was to gain insight into the ideal SCPs according to relevant stakeholder groups and to explore their motivations behind adding them. Patients composed a rather compact SCP, mainly focusing on category 2 on identification and treatment, including both information and support for themselves, with their motives being primarily based on their personal experiences and needs. HCPs and IT professionals constructed more comprehensive SCPs, with category 3 on oncological follow-up being the most important one, and HCPs additionally focusing on category 4 on *coordination.* When looking at their underlying motives, they all aligned with their respective areas of expertise: HCPs related their additions mainly to their roles as medical practitioners, such as providing a complete treatment plan and obtaining informed consent, while IT professionals’ contributions were mostly influenced by feasibility and privacy concerns.

In light of our findings and prior research focusing on other types of cancer (eg, colorectal and gynecological [23-25]) than melanoma, it is evident that patients place considerable emphasis on *identification and treatment of disease and therapy effects* (category 2), perceiving the SCP mainly as an informational and supportive tool. Thereby, patients’ unmet needs are related to their commonly faced challenges such as psychological distress, anxiety, depression, long-term and late effects, as well as the fear of recurrence. As a result, they express a need for a more comprehensive supportive care approach that includes nonmedical information, like peer support, financial guidance, return-to-work strategies, and psychological resource information [17,26-28]. Moreover, the emphasis on category 2 in our results underscores the importance of broadening traditional, current SCP content, which predominantly targets categories 1 and 3 on *information and education* and *oncological follow-up* [4]. Patients agreed that functions from category 2 (eg, psychosocial and societal support) are indeed essential for effective SSC, as it would both help them deal with certain issues and prevent searching for incorrect information online and related distress. Integrating (referrals to) this information in the SCP also allows all to be centralized in one place, instead of spread over paper-based information and web-based resources given to them by various HCPs. Despite patients’ agreement on category 2, their opinions differed on other functions. For example, some accentuated the need for general information (category 1, information and education) and more specifically on treatment effectiveness, but others felt that knowing this might negatively influence their hope toward a positive outcome. A reason for this may be found in patients’ motives for the composition of their ideal SCP, which were predominantly based on their personal, specific experiences with the disease and the knowledge they gained when they were affected and treated [29]. In addition, our findings align with previous research showing that providing certain information might be beneficial for some patients but can trigger fear among others [8,9], and thereby further underscore the importance of personalizing the SCP’s content [30,31]. To adequately tailor the SCP to each patient’s individual needs, future research should investigate ways for SCP tailoring, possibly through patient profile definition or integrating artificial intelligence (AI) methodologies. In addition, further quantitative research should follow to investigate the actual impact of our results on both SCPs’ implementation and effectiveness in clinical practice, for example, through a classic randomized controlled trial or partially randomized patient preference trial, the latter incorporating patient preferences in the process of randomization [32].

HCPs and IT professionals, in contrast, mainly based their ideal SCP preferences on their professional expertise. HCPs primarily related their choices to their clinical knowledge and roles as medical professionals and indicated what is needed to provide patients with a complete treatment plan and in order to obtain informed consent. IT experts offered more practical reasons, predominantly concerning feasibility and privacy issues, thereby including perspectives the end users may have insufficient knowledge about [29,33]. Both HCPs and IT professionals regarded *oncological follow-up (category 3)* as most important. Within this category, information about (tools for) detecting recurrences was added unanimously. This corresponds with previous literature that identified fear of recurrence as a prevalent psychological concern among melanoma survivors [6,17,34], and offering information and support concerning this has been shown to alleviate the intensity of such fears [35]. In the context of the ongoing digital health transformation and the workforce challenges in health care [36,37], AI offers promising solutions. AI could help in assessing skin abnormalities and thereby detecting recurrences in the future [38]. Although physicians have concerns about AI tools’ accuracy and potential health inequality risks, it could lead to fewer unnecessary consultations, cost reductions, and improved care pathways [39]. As a result, improving SSC practices regarding *oncological follow-up* could also facilitate advancements in category 4 on *care coordination*, a topic that HCPs in our study also deemed important. This suggests that the HCPs viewed the SCP as a care coordination tool, which could potentially address areas for improvement that were stressed in previous literature [40].

The feasibility and privacy concerns highlighted by IT professionals were particularly related to functions deemed vital to end users, ie, patients and HCPs, such as an overview of where to go (category 2), a personal follow-up schedule (category 3), and tools to improve information transmission between HCPs (category 4). Although legitimate, particularly given the patients’ unmet needs, it is important to investigate how to adequately address these issues. Suggestions put forth to mitigate some of these concerns included providing only the patients’ specialist’s name rather than their comprehensive contact information and linking the SCP to the electronic patient portal instead of incorporating a personal follow-up schedule directly within the SCP. However, since the use by HCPs in clinical practice and thereby the SCP’s implementation could be facilitated by linking it directly to the electronic health record [6], which has been shown feasible before [41], it should be investigated how to overcome these privacy concerns.

The above discussion suggests that HCPs, patients, and IT professionals attribute different but complementary roles to SCPs. Whereas HCPs view SCPs primarily as a coordination tool, patients stress their informational and supportive roles, and IT professionals see them as a data-sharing tool that must function in a safe and reliable manner. These differing perspectives can be explained by the organizational structure of melanoma care, including SSC, which is organized in networks with centralization of administration of systemic treatments across the Netherlands. In the Rijnmond Region, it operates from a “shared-care model” [42], where HCPs lead the network and its coordination while patients are positioned at the receiving end of care, thus receiving care, information, and support. From this point of view, the role of IT professionals in relation to SCPs is to ensure that coordination, information sharing, and support are conducted in a reliable manner.

### Recommendations for Future SCP Developers

The future SCP that we envision based on our results should address all the above roles, functioning both as a comprehensive information tool—facilitating the safe and reliable linking and sharing of information of multiple stakeholders through its digital aspect and connection with the patient’s electronic health record—and as a means to improve the coordination of melanoma care. Being better informed and supported will likely enhance patient empowerment, allowing them to take a more active role in managing their disease and treatment coordination and thereby facilitating shared decision-making [43,44], rather than merely being passive recipients of care. Thus, future developers should create SCPs that contain functions and features with both personal and general information, information about/referral to reliable information on psychosocial and societal support for patients, as well as information on lifestyle and tools for detecting recurrences, and with functions and features that facilitate care coordination. This SCP can and should be further personalized, depending on patients’ and HCPs preferences, by adding additional functions and features such as a personal follow-up schedule, information on where to go in case of questions [12], tips for informing relatives, and support for them.

### Strengths and Limitations

The main strength of this study is its inclusive approach to cocreation. Unlike many other cocreation studies that often engage only 1 stakeholder group, typically patients [45,46], or more recent at most 2 (patients and HCPs [47,48]), our study uniquely incorporated IT professionals. Whilst they are not the primary users of SCPs [4], their involvement proved invaluable as they offered crucial insights into the development process, particularly highlighting feasibility and privacy concerns. Moreover, we actively engaged all stakeholders from the inception of the SCP development rather than limiting their participation to its evaluation, as seen in, and of which the importance was stressed in, previous literature [7,33]. Furthermore, this cocreation approach aligns with the current shift toward value-based health care, endorsing the principles of patient partnership and shared decision-making [49]. Such an approach empowers the target audience and other stakeholders to shape the outcome actively, ensuring that the final SCP aligns closely with their needs and preferences. Moreover, the composition of our sessions with mixed groups fostered mutual learning and encouraged interactive discussions [13,14,50]. These elements collectively enhance the likelihood of the tool being widely accepted, facilitating more effective implementation and practical effectiveness.

A limitation of our study was its regional sample, which raises questions about the transferability of our findings. However, since melanoma care is uniformly organized in networks throughout the Netherlands, we expect that our results will be applicable outside our region and possibly to other countries if melanoma care is similarly organized. However, to reach an optimal, inclusive SCP, perspectives and needs of patients with varying levels of (health) literacy, socio-economic status, and backgrounds should also be investigated and incorporated. Another aspect warranting attention is the inclusion of an IT professional being a relative of a patient with melanoma. Even though we believe their experiences did not greatly affect the composition of IT professionals’ ideal SCP, it could be interesting to further investigate relatives’ perspectives on ideal SCPs and their underlying motivations for it. Based on previous literature, we know that relatives of cancer survivors can encounter significant challenges and have unmet needs throughout the patients’ disease trajectory [17,51,52] and are therefore sometimes even included in the definition of a cancer survivor [3]. Lastly, our participant pool only included patients who had finished their treatment for some time. Since retrospective experiences might differ from those of patients currently undergoing treatment, it is important to focus on this latter group in future research, ensuring a complete understanding of patients’ SSC needs throughout the whole disease trajectory.

### Conclusions

This cocreation study provides insights into stakeholders’ ideal melanoma SCP and the motivations behind them. Considering the diversity in both preferences and underlying motives regarding SCP composition between patients, HCPs, and IT specialists, it is crucial to develop a broad SCP that extends beyond traditional SCP content, emphasizing personalization. By understanding the motives and considerations of patients and HCPs in shaping their ideal SCPs, which we were able to elicit through the interaction and discussion between different stakeholders, thoughtful design can optimize patient care and support throughout the survivorship journey. At the same time, keeping the practical requirements of IT professionals in terms of feasibility and privacy in mind is important to ensure the ideal SCPs can be realized. In addition to continued stakeholder involvement, efforts should be focused on addressing the potential feasibility and privacy issues, particularly those related to personalization, to ensure the SCP meets the needs of both patients and HCPs.

## Appendix

Multimedia Appendix 1 Overview of all functions and features per category of survivorship care.

## Abbreviations

AI: artificial intelligence
COREQ: Consolidated Criteria for Reporting Qualitative Research
HCP: health care professional
SCP: survivorship care plan
SSC: survivorship care
