# Supplementary material for: Exploring Motives Behind Ideal Melanoma Survivorship Care Plans With Multiple Stakeholders: A Cocreation Study
Source: JMIR Cancer. 2025 Jan 2;11:e55746. doi: 10.2196/55746 (PMC11739727; doi:10.2196/55746)
Supplement: Multimedia Appendix 1 [file cancer_v11i1e55746_app1.docx]

| **Category of SSC** | **Functions** | **Features** |
| --- | --- | --- |
| Information and education | Personal information about the patient, including their disease and treatments | Information about the diagnosis, including eg, melanoma stage, Breslow thickness, which treatment 1 has had, is receiving or will receive |
|  | General information about the diagnosis, disease, treatments, and possible outcomes | Information about diagnosis (incl. explanations of stage and Breslow thickness, prognosis and symptom) |
|  |  | Information about what the treatment entails incl. treatment steps adapted to stage, treatment effectiveness and possible side effects, long-term effects and late effects |
|  |  | Information about the decision for (adjuvant) systemic therapy |
|  |  | Advice in the decision whether or not to go for (adjuvant) systemic therapy (incl. pros and cons) |
|  |  | Information about the possibility of a second opinion |
|  | Tips/information about informing relatives about the disease |  |
|  | Tips/information about informing about the disease at work |  |
| Identification and treatment | Psychosocial support | Tools to screen for psychological and social problems |
|  |  | Information about/referral to psychological and social support |
|  |  | Information about/referral to peer support |
|  | Societal support | Information about/referral to financial-, work-, and insurance-related support |
|  | Other supportive care | Information about and referral to physiotherapy and dietician |
|  | Family and caregiver support | Information about and referral to psychosocial support for family members and caregivers |
|  |  | Information about when to have family examined for skin abnormalities and why this is often not necessary |
|  | Overview of where to go in case of various questions and complaints |  |
| Oncological follow-up | Personal follow-up schedule | Patients’ personal follow-up schedule |
|  |  | Background information about follow-up (schedule), including information about the frequency, why this might differ per patient and between hospitals and why it stops |
|  | Information about and/or referral tot tools for detecting recurrences | Information about self-management in recognizing recurrence |
|  |  | Information about who to contact in case of changes of suspicions |
|  |  | Information about or referral to tools for detecting recurrences, eg, AI tools |
|  | Information about a healthy lifestyle | General information about a healthy lifestyle |
|  |  | Information about a healthy lifestyle specifically for patients with melanoma |
| Coordination | Information about/referral to a care coordinator, eg, case manager or fixed contact person |  |
|  | Tools to improve information transmission between HCPs |  |
